# Association of BMI trajectories and healthy aging among Chinese older adults: a national cohort study

**DOI:** 10.3389/fpubh.2025.1538261

**Published:** 2025-06-09

**Authors:** Xiaodan Kuang, Pan Ding, Liuhong Tian, Hongying Shi, Shize Wang

**Affiliations:** ^1^Department of Epidemiology and Health Statistics, School of Public Health, Wenzhou Medical University, Wenzhou, China; ^2^School of Mental Health, Wenzhou Medical University, Wenzhou, China; ^3^Development and Planning Office of Wenzhou Medical University (University-Local Cooperation Office), Wenzhou, China

**Keywords:** trajectory, body mass index, healthy aging, LCGM, variability

## Abstract

**Objectives:**

We aimed to explore the association between body mass index (BMI) trajectories and subsequent healthy aging in Chinese older adults.

**Methods:**

A prospective, population-based cohort study. Older adults (≥60 years) from the Chinese Longitudinal Health and Longevity Survey with three measurements of BMI (2008, 2011, 2014) were eligible for follow-up. Healthy aging was evaluated in 2018 and was defined as being free of major chronic diseases, no physical limitations, no cognitive impairment, and good mental health. We identified BMI trajectories by latent class growth modeling and explored their association with healthy aging by logistic regression model.

**Results:**

Of 2,698 older Chinese (median [IQR] age: 78.00 [71.00, 86.00] years; female: 51.82%), 518 (19.20%) participants had reached healthy aging after follow-up. Three BMI trajectories were identified: low-normal stable (49.30%), normal slight increase (43.07%), and overweight to obesity (7.64%). Compared with the normal slight increase trajectory, the overweight to obesity trajectory had significantly lower odds of healthy aging (*OR* = 0.57, 95%*CI*: 0.36, 0.93). Interestingly, among the four dimensions of healthy aging, the low-normal stable trajectory was associated with lower odds of no cognitive impairment, and the overweight to obesity trajectory was associated with lower odds of no major chronic diseases. Additionally, higher BMI variability was associated with lower odds of healthy aging independent of baseline BMI.

**Conclusions:**

Maintaining weight to stave off the transition from overweight to obesity is crucial for healthy aging among older Chinese. The findings underscore the critical importance of monitoring dynamic changes in BMI in older Chinese adults.

## Introduction

The global population is aging at an accelerating rate, with the proportion of people over the age of 60 will increase from 1 billion (12%) in 2015 to 2.1 billion (22%) in 2050 (1). In China, by the end of 2023, the proportion of the population aged 60 and above has reached 21.1%, and diversified health problems of older adults led to increase economic burden, aging intensifies has become a public health problem (2, 3). The United Nations's 2020 World Report proclaimed 2021–2030 as the “Decade of Healthy Aging” (4). The concept of “healthy aging” (or “successful aging”) is high cognitive and physiological functioning, low risk of disease and disability, and active engagement in life according to Rowe and Kahn's framework model in 1987 (5). The average rate of healthy aging was 35.8% (6), China only has a rate of 15.7%, whereas East Asian countries such as South Korea and Japan have rates of 25.5% and 29.2%, respectively (7). To effectively promote the sound development of an aging society, attention has been directed toward key variables influencing healthy aging including moderate exercise, a nutritious diet, maintenance of a healthy weight, and other lifestyle habits (8).

Body mass index [BMI, calculated as weight (kg) divided by height squared (m^2^)] is a common indicator of body fat and fitness in adults and is associated with mortality, specific diseases and health conditions, but in older adults, relying on a single-point BMI measurement is not sufficient to capture the long-term effects of body size on health outcomes as BMI can fluctuate over time (9). In recent years, many studies on the BMI trajectories of older adults have shown that chronically overweight or obesity, continue to increase after becoming overweight, are at increased risk of developing health problems such as hypertension, diabetes, cardiovascular disease, cognitive impairment, and frailty compared with those in the normal weight stable group (10–12). However, the association between BMI trajectories in older adults (≥60 years) and overall health assessed using multidimensional structures is unclear.

In this cohort study, we aimed to identify the trajectories of BMI among Chinese older adults and to explore their association with subsequent healthy aging. Since fluctuations in BMI within the normal range are a normal physiological phenomenon, we hypothesized that participants from overweight to obesity would have a lower rate of healthy aging compared to those with the normal stable BMI.

## Methods

### Study design and participants

We used data from the Chinese Longitudinal Health and Longevity Survey (CLHLS), an on-going national representative prospective cohort study of Chinese individuals aged ≥60 years, covering approximately 85% of the population in China (13, 14). Since 1988, CLHLS has been gathering demographic, lifestyle, and health information every 2–3 years through face-to-face interviews conducted by trained health workers from local Centers for Disease Control and Prevention and community hospitals (14). Details about CLHLS have been described elsewhere (13). CLHLS is considered to be of high quality because of its few missing data, high response rate, low dropout rate (average per wave was 4.85%), and compatibility with other countries' data (15). The CLHLS was approved by the Research Ethics Committee of Peking University (IRB00001052-13074), and the written informed consent of all participants were obtained.

BMI variables were collected from 2008, so a total of 5,245 participants (≥60 years) surveyed in 2008, 2011, and 2014 were eligible for this study. We excluded 1,158 participants with five major chronic diseases at baseline (8, 16, 17), 353 participants with missing weight data in any one of these three surveys, 809 participants without follow-up data after 2014, and 227 participants with missing healthy aging data (2018), leaving 2,698 eligible participants for this study (eFigure 1). To assess the possibility of potential selection bias, we compared the included participants (*n* = 2,698) with those excluded due to the lack of follow-up or healthy aging data (*n* = 1,036) and found that the basic characteristics were largely comparable between these included and excluded participants, except that excluded participants were more likely to be from urban areas (eTable 1). This study followed the Strengthening the Reporting of Observational Studies in Epidemiology (STROBE) reporting guidelines.

### Exposure assessment

Weight and height of all participants were measured three times in 2008, 2011, and 2014. It was measured by trained medical staff using standardized programs. BMI is calculated by dividing the weight (kg) by the square of the height (m). According to the recommended standards of adults in China (18), body type was divided into underweight (<18.5 kg/m^2^), normal (18.5–23.9 kg/m^2^), overweight (24.0–27.9 kg/m^2^), and obesity (≥28.0 kg/m^2^). Based on the BMI in 2008, 2011, and 2014, we identified BMI trajectories using Latent Class Growth Modeling (LCGM) (19). In the secondary analysis, we used the standard deviation of BMI from three wave surveys to evaluate the variability of BMI.

### Assessment of healthy aging

Based on previous research and the framework of Rowe and Kahn's concepts (5, 16), healthy aging was evaluated in 2018 and was defined as having no five major chronic diseases (cancer, chronic lung disease, diabetes, heart disease and stroke), no cognitive impairment (assessed by the Chinese version of Mini-Mental State Assessment (MMSE) and education-stratified cutoffs), no physical limitations [evaluated by the Katz Index of Independence in Activities of daily living (ADL)] and good mental health [no depression (Center for Epidemiologic Studies Depression Scale, CESD-10) or anxiety (Generalized Anxiety Disorder Scale, GAD-7)]. If any one of the criteria was not met, it was defined as usual aging. Detailed descriptions of each of the 4 domains of healthy aging are listed in Table 1 (7, 20–29).

**Table 1:** Definitions and dimensions of healthy aging.

**Dimensions**	**Assessments**	**Definitions**
**Absence of major chronic diseases**	According to relevant research (7, 20), five major chronic diseases that cause the burden of diseases in the older Chinese were cancer, chronic lung disease, diabetes, heart disease and stroke. Participants were asked “Do you suffer from the following chronic diseases now?” and “Have you been diagnosed with any disease by the hospital?”. One of these two questions was answered “yes”, he was considered to have this disease.	No major disease was defined as participants not reporting any of the five chronic diseases.
**No cognitive impairment**	Cognitive function was measured by the Chinese version of Mini-Mental State Assessment (MMSE) (21), which had 25 items with a total of 30 points in five parts: general ability, reaction ability, attention and calculation ability, recall ability, language understanding and self-coordination ability. The higher the score, the better the level of cognitive function. Chinese study (22) found that the cut-off point of MMSE was different according to the educational level of the interviewees, and a large proportion of illiterate older people (57.20%) were observed in our study.	We adopt the criteria recommended by Shanghai Mental Health Center to define cognitive impairment (23, 24): the total score of people without formal education is <18, those with 1–6 years of education is <21, and those with more than 6 years of education is <25.
**No physical limitations**	We used the Katz Index of Independence in Activities of daily living (ADL) (25) to evaluate physical function, including dressing, bathing, feeding, indoor transferring, toileting, and continence.	Physical limitations were defined as at least one task that required assistance from others (26).
**Good mental health**	In the CLHLS 2018 questionnaire, mental health assessment included two parts: Depression Scale and Anxiety Scale (20). Depression was assessed using Center for Epidemiologic Studies Depression Scale (CESD-10)(20), people were asked about the frequency of various emotions in the past two weeks (7 positive questions and 3 reverse questions). The score of depression ranges from 0 to 30 points, with higher scores indicating more serious depression. CLHLS used seven items of Generalized Anxiety Disorder Scale (GAD-7)(27) to assess anxiety, people were asked about the frequency of seven core symptoms of GAD in the past 2 weeks and the total score of this scale is 0–21.	The total scores ≥10 (28) was considered in a depressive state. GAD-7 ≥10 (29) was defined as anxiety. Participants with no depression and no anxiety were considered as having good mental health.
**Healthy aging**	Healthy aging was defined as absence of major chronic diseases, no cognitive impairment, no physical limitations and have good mental health.	

### Assessment of potential covariates

The covariate information was collected in 2008 based on previous studies (15). Demographic characteristics included age, gender (male, female), ethnicity (Han Chinese, others) (30), residence district (city, town, rural), education (illiteracy, primary school and below, junior high school and above), marital status (15) (married and living with spouse, others), living expenses (adequate, inadequate), and occupation (farmer, others). Lifestyle information included sleep quality (good, fair, poor), sleep duration (31) (<7, 7–8, >8 h/day), smoking status (never, former, current), drinking status (never, former, current), exercise status (never, former, current) and dietary diversity (Dietary Diversity Score, DDS), and BMI at baseline (underweight, normal, overweight, and obesity). If the participant was unable to answer, then the questions (except those that had to be filled out by the participant) could be answered by an adult family member who was most familiar with the participant's living situation.

When the covariates in 2008 were missing (variables are missing in <5% of the participants), we used the data collected in 2011 or 2014 to impute them. Finally, the proportion of missing values in covariates was 0.07% (missing values for occupation and education were 2 and 2, respectively).

### Statistical analyses

We used Latent Class Growth Modeling (LCGM) (19) (R [lcmm package]) to identify subgroups with similar latent BMI trajectories in three waves. Generally, we fitted the longitudinal BMI data into the model with a mixture of multiple latent trajectories by the maximum likelihood method (17). We implemented linear, quadratic and cubic models and used the Bayesian Information Criterion (BIC) and entropy to identify the optimal number and shapes of trajectory groups (eTable 2), and the proportion of participants in each trajectory should meet the condition of at least 5% of the population (32). Then, the posterior probabilities computed via Bayes' theorem were used to test the identification ability, with detailed methods found in the LCGM tutorial (19, 33).

The Chi-square test was used to compare differences in categorical variables and Kruskal–Wallis test in ordinal data and non-normal continuous data. We used logistic regression models to estimate the odds ratios (*ORs*) and 95% confidence intervals (*CIs*). Model I was adjusted for baseline age, gender. Model II was additionally adjusted for residence district, ethnic group, educational level, marital status, living expenses and occupation. Model III was additionally adjusted for lifestyle factors, including smoking history, drinking status, exercise, dietary diversity, sleep quality, and sleep duration. Model IV was additionally adjusted for body mass index at baseline. To explore the stability and heterogeneity of the associations between different BMI trajectories with healthy aging in different lifestyle situations, we further performed stratified analyses by sex, age and lifestyle (smoking, drinking, exercise, diet, sleep quality and sleep duration). We used the likelihood ratio test to evaluate the statistical significance of the multiplicative interaction between lifestyles and BMI trajectories.

To compare with previous studies, we conducted a secondary analysis utilizing 2014 BMI data to investigate its correlation with healthy aging and its various dimensions through the application of restricted cubic spline curves and threshold effect analysis. For verifying the robustness of the results, we conducted a series of sensitivity analyses, the specific methods are presented in Supplementary data (Appendix 1).

All statistical analyses were performed using Empower (R) (https://www.empowerstats.net/cn/, X & Y solutions, Inc. Boston MA) and R (https://www.r-project.org/). All *P*-values were 2-sided and *P* < 0.05 was considered statistically significant.

## Results

The median (IQR) age of the 2,698 older adults was 78.00 (71.00–86.00) years, and 51.82% of them were female. After the four-year follow-up period from 2014 to 2018, 928 (34.40%) had no five major chronic diseases, 1,249 (46.29%) had no physical limitations, 994 (36.84%) had good mental health, and 1,296 (48.04%) had no cognitive dysfunction. A total of 518 participants (19.20%) had reached healthy aging, while the rest were normal aging, and 1,051 of them died during this period.

### Trajectories of BMI in older adults from 2008 to 2014

**Figure 1:**
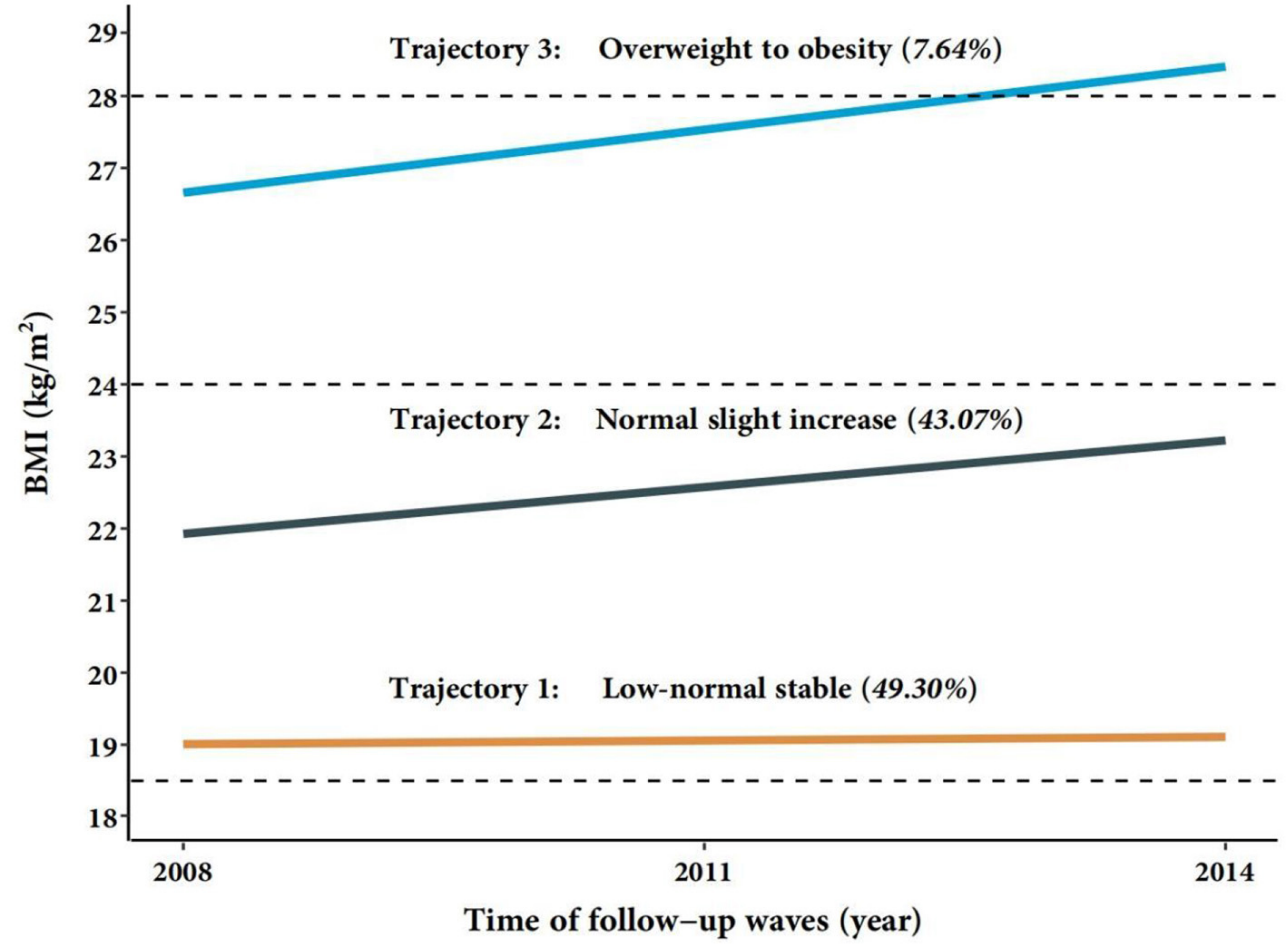
Trajectories of BMI in the Chinese order adults from 2008 to 2014. BMI, body mass index. Low-normal stable (*n* = 1,330); Normal slight increase (*n* = 1,162); Overweight to obesity (*n* = 206); Total (*n* = 2,698).

Three different BMI trajectories in older adults from 2008 to 2014 were identified (eTable 2): the average BMI of 1,330 (49.30%) participants maintained around 19 kg/m^2^ (low-normal stable trajectory), 1,162 (43.07%) participants had a moderate increase in BMI from 21.77 kg/m^2^ to 23.17 kg/m^2^ (normal slight increase trajectory), and 206 (7.64%) participants had an increase in BMI from overweight to obesity (overweight to obesity trajectory) (Figure 1). We calculated the posterior probability of each individual being assigned to each trajectory group and assigned it to the trajectory group with the highest posterior probability. The average posterior probabilities for each trajectory groups were >0.70 (0.853, 0.888, and 0.903, respectively), suggesting that each trajectory we fitted had high internal reliability and was sufficiently distinguishable among participants with different BMIs.

### Baseline characteristics of participants

Table 2 showed the age-adjusted baseline characteristics of participants with different BMI trajectories. Compared with the normal slight increase trajectory, participants with the overweight to obesity trajectory were slightly younger, tended to be female, non-farmers, had exercise, and had better dietary diversity. Conversely, participants with the low-normal stable trajectory were slightly older, more likely to be female, illiterate, farmer, living alone, non-smokers, non-drinkers, non-exercisers, had worse sleep quality, and had worse dietary diversity. All *P* < 0.05.

**Table 2:** Age-adjusted baseline characteristics of participants by BMI trajectories in the CLHLS study^a^ (*n* = 2,698).

	**BMI trajectories**			
**Characteristic**	**Low-normal stable (*****n** **=*** **1,330)**	**Normal slight increase (*****n** **=*** **1,162)**	**Overweight to obesity (*****n** **=*** **206)**	***P-*** **value**
Age, years, median (IQR)^b^	81.00 (73.00–89.00)	76.00 (69.00–84.00)	72.00 (67.00–78.00)	**<0.001**
BMI in 2008, kg/m^2^, mean (SD)	18.90 (2.30)	21.93 (2.47)	26.80 (3.27)	**<0.001**
BMI in 2011, kg/m^2^, mean (SD)	18.87 (2.27)	22.92 (2.38)	27.81 (2.81)	**<0.001**
BMI in 2014, kg/m^2^, mean (SD)	18.94 (2.16)	23.31 (2.31)	28.61 (2.85)	**<0.001**
Female, *n* (%)	754 (58.16)	533 (46.42)	111 (54.25)	**<0.001**
Ethnicity, Han, *n* (%)	1,204 (90.5)	1,092 (93.86)	198 (95.79)	**0.001**
Education level, *n* (%) ^c^				**<0.001**
Illiteracy	799 (62.2)	572 (50.99)	97 (48.38)	
Primary school and below	431 (30.89)	441 (36.92)	78 (37.27)	
Junior high school and above	99 (6.91)	148 (12.08)	31 (14.35)	
Residential district, *n* (%)				**<0.001**
City	84 (6.42)	128 (11.19)	33 (15.69)	
Town	272 (20.61)	262 (22.62)	48 (22.94)	
Rural	974 (72.97)	772 (66.19)	125 (61.37)	
Living expenses, adequate, *n* (%)	996 (74.93)	923 (79.57)	184 (89.04)	**<0.001**
Farmer, *n* (%) ^c^	1,106 (83.08)	842 (72.64)	130 (63.64)	**<0.001**
Marital status, *n* (%)				**<0.001**
Married and living with spouse	537 (37.63)	644 (53.01)	126 (59.02)	
Others	793 (62.37)	518 (46.99)	80 (40.98)	
Smoking status, *n* (%)				**0.001**
Never	881 (67.41)	696 (60.27)	136 (66.1)	
Former	137 (10.27)	164 (14.18)	32 (15.77)	
Current	312 (22.33)	302 (25.55)	38 (18.12)	
Drinking status, *n* (%)				**<0.001**
Never	924 (70.08)	693 (60.03)	124 (59.96)	
Former	142 (10.69)	151 (13.15)	31 (15.3)	
Current	264 (19.22)	318 (26.82)	51 (24.73)	
Exercise status, *n* (%)				**<0.001**
Never	848 (63.98)	695 (59.72)	97 (47.09)	
Former	118 (8.86)	90 (7.85)	17 (8.58)	
Current	364 (27.15)	377 (32.43)	92 (44.34)	
Sleep quality, *n* (%)				**0.032**
Good	866 (65.33)	819 (70.52)	150 (73.1)	
Fair	333 (24.91)	240 (20.6)	38 (18.52)	
Bad	131 (9.76)	103 (8.88)	18 (8.38)	
Sleep duration, hours, *n* (%)				0.689
<7	365 (27.08)	300 (25.9)	50 (23.87)	
7–8	551 (40.88)	512 (43.37)	89 (43.06)	
>8	414 (32.03)	350 (30.74)	67 (33.07)	
Dietary diversity, good, *n* (%)	921 (68.92)	876 (74.87)	174 (84.34)	**<0.001**

BMI, body mass index; SD, standard deviation.

The value of a continuous variable is the mean (SD), a categorical variable is the *n* (%). The Chi-square test was used for comparing unordered categorical data, the analysis of variance was used for normal variables and the Kruskal–Wallis test was used for ordinal data and skewed continuous data, and bold values indicated statistical significance *P* < 0.05.

^a^Data were standardized according to the age distribution (continuous) of the study population at baseline.

^b^Value was not age adjusted.

^c^The missing values for occupation and education were 2 and 2, respectively (n = 2,696).

### Association between different BMI trajectories and healthy aging in older adults

Table 3 summarized the *ORs* of healthy aging and its four dimensions associated with BMI trajectories. Compared with the normal slight increase trajectory, the overweight to obesity trajectory was significantly associated with lower odds of healthy aging after adjustment for potential confounders. The multivariable-adjusted *ORs* for Model 4 were 0.90 (95%*CI*: 0.70, 1.15) for the low-normal stable trajectory and 0.57 (95%*CI*: 0.36, 0.93) for the overweight to obesity trajectory. The association between BMI trajectories and the four dimensions of healthy aging differed. Compared with the normal slight increase trajectory, older adults in the overweight to obesity trajectory had a multivariable-adjusted *OR* of 0.46 (95%*CI*: 0.31, 0.69) for being free of chronic disease. In contrast, those in the low-normal stable trajectory had a multivariable-adjusted *OR* of 0.70 (95%*CI*: 0.56, 0.87) for no cognitive impairment.

**Table 3:** *ORs (95%CIs)* of healthy aging and its four domains by BMI trajectories in the CLHLS Study.

**Outcome**	**No**.	**Healthy aging, *n* (%)**	**Model l^a^**	**Model II^b^**	**Model III^c^**	**Model IV^d^**
**Healthy aging by BMI trajectories**						
Low-normal stable	1,330	215 (16.17)	0.93 (0.75, 1.16)	0.91 (0.73, 1.13)	0.90 (0.72, 1.12)	0.90 (0.70, 1.15)
Normal slight increase	1,162	260 (22.38)	1.00 [Reference]	1.00 [Reference]	1.00 [Reference]	1.00 [Reference]
Overweight to obesity	206	43 (20.87)	**0.66 (0.45, 0.96)**	**0.66 (0.45, 0.97)**	**0.67 (0.46, 0.99)**	**0.57 (0.36, 0.93)**
**Free of main chronic diseases by BMI trajectories**						
Low-normal stable	1,330	429 (32.26)	0.99 (0.83, 1.18)	0.96 (0.80, 1.15)	0.96 (0.80, 1.14)	0.94 (0.77, 1.14)
Normal slight increase	1,162	434 (37.35)	1.00 [Reference]	1.00 [Reference]	1.00 [Reference]	1.00 [Reference]
Overweight to obesity	206	65 (31.55)	**0.60 (0.43, 0.83)**	**0.63 (0.45, 0.87)**	**0.62 (0.45, 0.87)**	**0.46 (0.31, 0.69)**
**No physical limitations by BMI trajectories**						
Low-normal stable	1,330	529 (39.77)	0.95 (0.78, 1.14)	0.93 (0.77, 1.13)	0.92 (0.76, 1.11)	0.82 (0.66, 1.01)
Normal slight increase	1,162	598 (51.46)	1.00 [Reference]	1.00 [Reference]	1.00 [Reference]	1.00 [Reference]
Overweight to obesity	206	122 (59.22)	0.85 (0.60, 1.19)	0.88 (0.63, 1.24)	0.88 (0.63, 1.24)	0.97 (0.64, 1.46)
**Good mental health by BMI trajectories**						
Low-normal stable	1,330	396 (29.77)	**0.78 (0.65, 0.93)**	**0.82 (0.68, 0.98)**	**0.83 (0.69, 1.00)**	0.86 (0.70, 1.06)
Normal slight increase	1,162	495 (42.60)	1.00 [Reference]	1.00 [Reference]	1.00 [Reference]	1.00 [Reference]
Overweight to obesity	206	103 (50.00)	0.95 (0.69, 1.31)	0.90 (0.65, 1.25)	0.90 (0.65, 1.24)	0.78 (0.53, 1.16)
**No cognitive impairment by BMI trajectories**						
Low-normal stable	1,330	517 (38.87)	**0.73 (0.61, 0.89)**	**0.74 (0.61, 0.90)**	**0.74 (0.61, 0.90)**	**0.70 (0.56, 0.87)**
Normal slight increase	1,162	642 (55.25)	1.00 [Reference]	1.00 [Reference]	1.00 [Reference]	1.00 [Reference]
Overweight to obesity	206	137 (66.50)	0.98 (0.69, 1.40)	0.98 (0.69, 1.40)	0.98 (0.69, 1.41)	0.88 (0.57, 1.36)

BMI, body mass index (calculated as weight (kg) divided by height (meters) squared); OR, odds ratio; CI, confidence interval.

Bold values indicated statistical significance *P* < 0.05.

^a^Model I was adjusted for age (continuous), gender (male, female).

^b^Model II was additionally adjusted for residence district (city, town, rural); ethnicity (Han, others); educational level (illiteracy, primary school and below, junior high school and above); marital status (married and living with spouse, others); living expenses (adequate, inadequate); occupation (farmer, others).

^c^Model III was additionally adjusted for lifestyle factors, including smoking history (never, former, current); drinking status (never, former, current); exercise (never, former, current); dietary diversity (good, bad); sleep quality (good, fair, bad) and sleep duration (<7, 7-8, >8 hours).

^d^Model IV was additionally adjusted for body mass index at baseline (underweight, normal, overweight, obesity).

**Figure 2:**
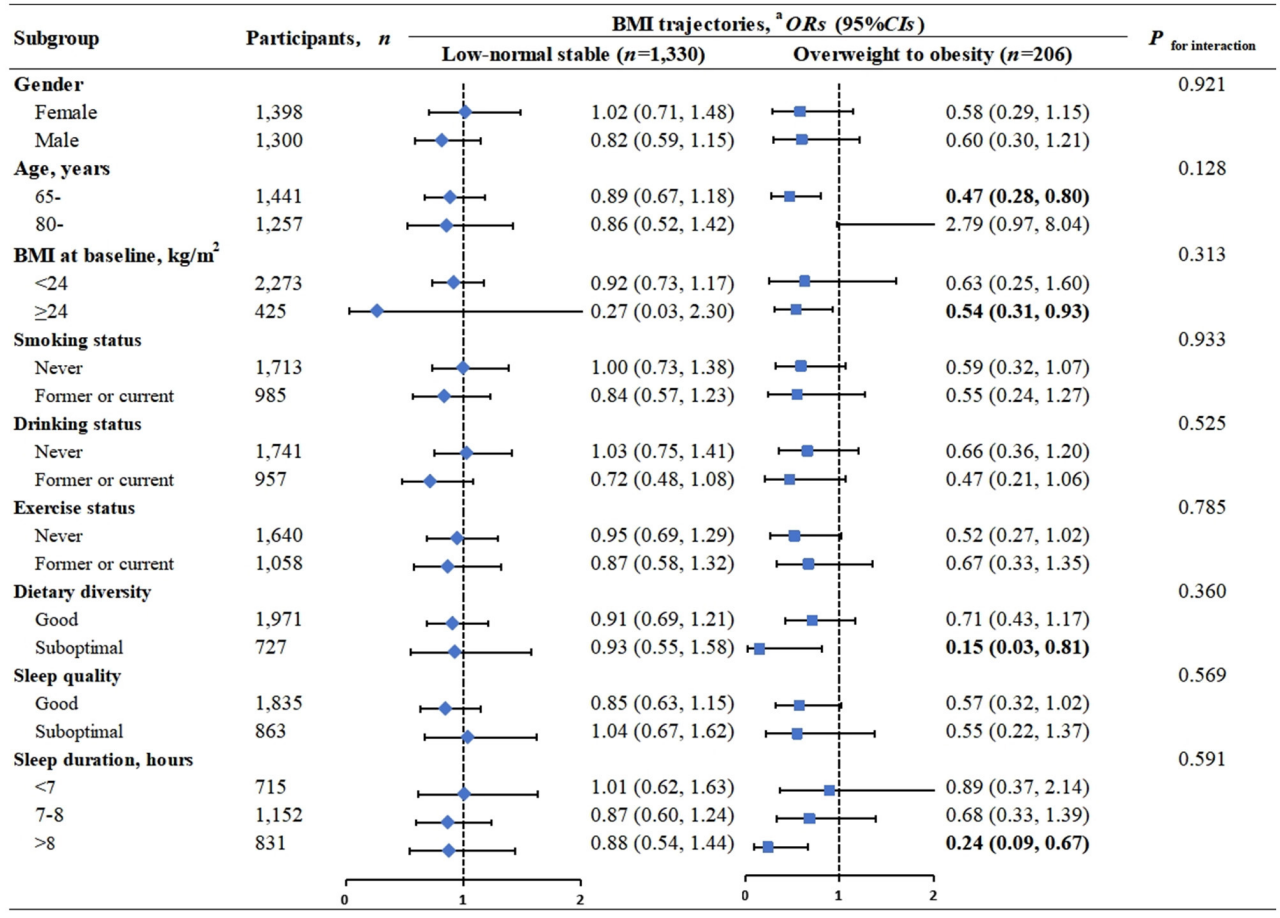
Association of healthy aging with BMI trajectories, stratification analyses. BMI, body mass index (calculated as weight (kg) divided by height (meters) squared); *OR*, odds ratio; *CI*, confidence interval. The Normal slight increase trajectory (*n* = 1,162) is included as the reference group. Bold values indicated statistical significance *P* < 0.05. ^a^Adjusted for age (continuous), gender (male, female); residential district (city, town, rural); ethnic group (Han, others); educational level (illiteracy, primary school and below, junior high school and above); marital status (married and living with spouse, others); living expenses (adequate, inadequate); occupation(farmer, others); and lifestyle factors, including smoking history (never, former, current); drinking status (never, former, current); exercise(never, former, current); dietary diversity (good, bad); sleep quality (good, fair, bad) and sleep duration (<7, 7–8, >8 h), and BMI at baseline (underweight, normal, overweight, obesity), except for the stratification factor itself.

In the stratified analyses, the associations between BMI trajectories and healthy aging were consistent among subgroups of different gender, age, BMI, lifestyles including smoking, drinking, exercise, diet, sleep quality and sleep duration, all *P* for interaction >0.05 (Figure 2). Specifically, in almost every subgroup, the overweight to obesity trajectory was consistently associated with lower odds of healthy aging.

Based on the results of Appendix 2, Table 4, and eTable 3, we discovered our findings remained robust in several sensitivity analyses.

**Table 4:** Sensitivity analyses of the association of healthy aging with BMI trajectories.

		**BMI trajectories**, ***ORs*** **(95%*****CIs*****)**		
**Outcomes**	***n***	**Low-normal stable (*****n** **=*** **1,330)**	**Normal slight increase (*****n** **=*** **1,162)**	**Overweight to obesity (*****n** **=*** **206)**
**1**. ^a^**Exclusion**				
Exclusion of participants who died within 2014–2018	1,647	1.03 (0.79, 1.34)	1.00 [Reference]	**0.57 (0.34, 0.95)**
**2**. ^a^**Limiting population to:**				
Farmers	2,078	0.86 (0.66, 1.13)	1.00 [Reference]	**0.36 (0.19, 0.68)**
Han Chinese	2,494	0.98 (0.76, 1.26)	1.00 [Reference]	**0.52 (0.32, 0.86)**
Illiterate	1,468	1.03 (0.71, 1.49)	1.00 [Reference]	**0.39 (0.18, 0.86)**
Adequate living expenses	2,100	1.01 (0.76, 1.33)	1.00 [Reference]	**0.58 (0.35, 0.97)**
**3. Further adjustment**				
Self-reported health status^b^	2,698	0.90 (0.70, 1.15)	1.00 [Reference]	**0.59 (0.36, 0.95)**
Hypertension^c^	2,698	0.88 (0.69, 1.13)	1.00 [Reference]	**0.59 (0.36, 0.95)**
**4**. ^a^**Alternative definitions of healthy aging**				
Add leisure activities in dimensions of healthy aging	2,698	0.81 (0.62, 1.04)	1.00 [Reference]	**0.61 (0.38, 0.99)**
Add IADL in four dimensions of healthy aging	2,698	0.78 (0.57, 1.08)	1.00 [Reference]	0.58 (0.32, 1.03)

OR, odds ratio; CI, confidence interval; BMI, body mass index (calculated as: weight (kg) divided by height (meters) squared); IADL, Instrumental Activities of Daily Living. Bold values indicated statistical significance *P* < 0.05.

^a^Adjusted for age (continuous), gender (male, female); residential district (city, town, rural); ethnic group (Han, others); educational level (illiteracy, primary school and below, junior high school and above); marital status (married and living with spouse, others); living expenses (adequate, inadequate); occupation (farmer, others); and lifestyle factors, including smoking history (never, former smoker, or current smoker); drinking status (never, former, or current); exercise (never, former, or current); dietary diversity (good, bad); sleep quality (good, fair, bad) and sleep duration (<7, 7–8, >8 h), and BMI at baseline (underweight, normal, overweight, obesity).

^b^Self-reported health status is divided into two categories: good or suboptimal.

^c^Hypertension is divided into two categories: yes or no.

### Joint associations of BMI trajectories and variability of BMI with healthy aging

**Figure 3:**
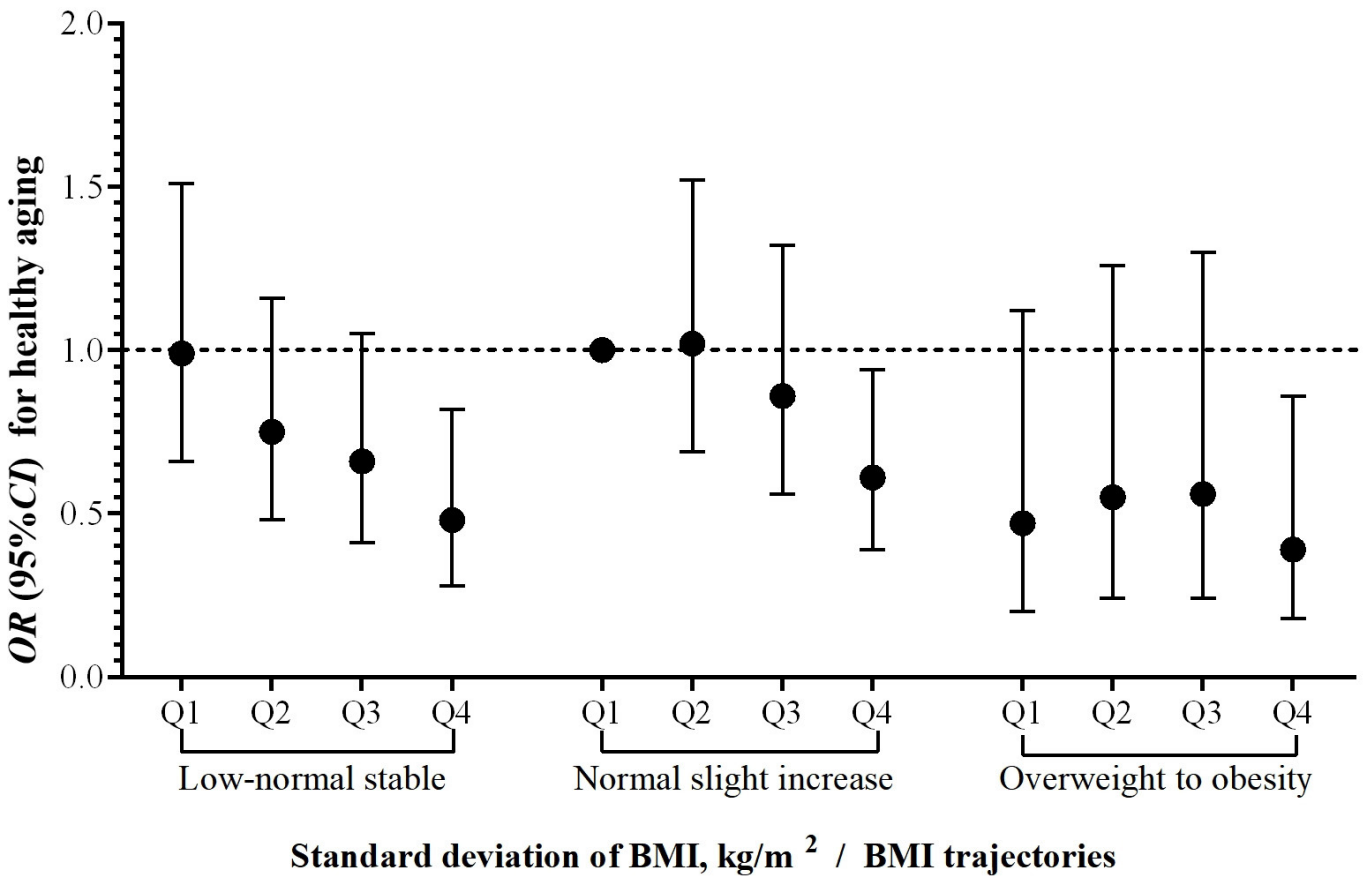
Joint effects of BMl trajectories and variability of BMl on healthy aging. **Abbreviations** BMI, body mass index (calculated as weight (kg) divided by height (meters) squared); OR, odds ratio; CI, confidence interval. Standard deviation of BMI was divided into four groups using quartiles (Q1–Q4). The **horizontal dashed line** indicates an *OR* of 1.00; the **vertical error bars** represent 95% *CI*s. Adjusted for age (continuous), gender (male, female); residential district (city, town, rural); ethnic group (Han, others); educational level (illiteracy, primary school and below, junior high school and above); marital status (married and living with spouse, others); living expenses (adequate, inadequate); occupation (farmer, others); and lifestyle factors, including smoking history (never, former smoker, or current smoker); drinking status (never, former, or current); exercise (never, former, or current); dietary diversity (good, bad); sleep quality (good, fair, bad) and sleep duration (<7, 7–8, >8 h), and BMI at baseline (underweight, normal, overweight, obesity).

Appendix 2 and Figure 3 indicated that compared to individuals with a normal slight increase trajectory and the least BMI variability, the odds of healthy aging were lowest among those with an overweight to obesity trajectory and a BMI standard deviation in the fourth quartile (OR = 0.39, 95%*CI*: 0.18, 0.86). Higher BMI variability is an important marker of healthy aging independent of baseline BMI.

### Association between BMI in 2014 and healthy aging and its four dimensions

In comparison to older adults with a normal weight range (BMI = 18.5–23.9 kg/m^2^), being underweight (BMI <18.5 kg/m^2^) poses a risk to mental health and cognitive function, while obesity (BMI ≥ 28 kg/m^2^) is associated with risk for chronic disease but is considered a protective factor for cognitive function. Restrictive cubic spline plots demonstrated an inverted U-shaped correlation between BMI and all three dimensions of healthy aging except for cognitive function (eTable 5; eFigure 2).

## Discussion

### Main findings

We identified three BMI trajectories from 2008 to 2014 in this cohort study and found that the BMI trajectory of most Chinese older people was stable at a normal or low-normal level. Compared with participants with a normal slight increase trajectory, those with overweight to obesity trajectory had significantly lower odds of healthy aging, and this association was more pronounced on the chronic disease dimension. We also observed a linear negative association between the variation (standard deviation) in BMI and healthy aging. Moreover, compared to participants with normal slight increase trajectory and the least BMI variability, the subsequent healthy aging rate is significantly reduced for those with the overweight to obesity trajectory and with a large variation in BMI.

### Comparison with other studies and explanations

This study used three repeated measurements to model BMI trajectories during older adulthood among Chinese older adults, an approach validated in previous studies (19, 34, 35). Existing studies in older adults (36–38), further confirms that this measurement frequency are sufficient to model BMI trajectories in this population. The trajectory analysis revealed that the BMI trajectories of most Chinese older adults tend to stay within the normal weight range (BMI: 18.5–23.9 kg/m^2^), which was consistent with the survey results in Taiwan (11). However, the distribution of BMI trajectories in older adults differed by region. An early study of the BMI trajectory in the United States found that 70%-80% of middle-aged and older people were overweight and obesity (39). The first survey conducted in Asia on the BMI trajectory (38) found that BMI for older Japanese was mainly within the normal weight range (BMI: 18.5–25.0 kg/m^2^), which is also similar to our results. Such differences may be attributed to racial differences, lifestyle and dietary habits. A study involving the analysis of BMI trajectories in older adults showed significant racial differences among Black, White, and Hispanic individuals (39). Therefore, it is recommended to focus on BMI trajectories between different populations and further study the BMI trajectories and their changing mechanisms in Asian and non-Asian older adults.

To the best of our knowledge, this is the first prospective study to investigate the association between BMI trajectories and healthy aging in older adults. Many current studies had focused on BMI and individual health outcomes among adults, and found that having a high BMI was related to chronic diseases (8), psychological problems (40), cognitive impairment (41) and disability (9, 26). A Canadian study (10) showed that individuals in the Overweight-Stable, Obesity I-Stable, and Obesity II -Stable groups experienced more chronic diseases, cognitive impairment, and declines in self-rated overall health than those in the Normal-Stable group. Therefore, our study combines the four aspects of chronic diseases, cognitive health, mental and physical function to evaluate healthy aging, which provides more concrete evidence for the association between BMI trajectories and the overall health of older adults. Previous studies commonly used frailty as the primary outcome, for example, studies conducted in the USA (42), Finland (43), Taiwan (China) (11) have shown that low and very high BMI are associated with a higher risk of frailty development in middle-aged and older people. Although frailty and healthy aging were defined differently, they are both an overall indicator that can measure the health and quality of life of older people. Thus, the findings of our study are similar to these findings, in which we observed that individuals in the overweight to obesity trajectory were significantly associated with reduced rates of healthy aging compared with those in the normal slight increase trajectory.

According to a literature review, some cross-sectional studies have shown a significant association between lower body weight or BMI and successful aging (44, 45). In our study, this result is not significant but a similar trend exists. Similar to our findings, several studies showed that overweight was significantly associated with a shorter healthy life expectancy (20). For example, a higher BMI in midlife was associated with significantly lower odds of healthy aging in French (8), and relevant research in China (46) showed that preventing central obesity and avoiding BMI increases were beneficial to the successful aging of older adults, which is consistent with our research results. Therefore, our study supplements evidence on BMI trajectories and healthy aging in older adults.

The impact mechanisms of BMI on healthy aging are complex. Firstly, the increase in body fat (which comes with a high BMI) is what brings about metabolic consequences due to its inflammatory effects (such as cytokine release, adipocyte death, and macrophage infiltration) in adipose tissue (47), along with high leptin (due to the increase in fat mass itself) (48). This inflammatory process generates insulin resistance, elevated blood glucose, and, in turn, increased sorbitol levels, which reduce anti-inflammatory molecules (like glutathione), producing fewer vasodilators (49). This exposure leads to greater susceptibility to atherosclerotic processes (17). Secondly, an increase in pro-inflammatory adipocytokines secreted by adipose tissue may lead to low-grade systemic inflammation, inducing various chronic diseases (8, 11). Higher body weight, especially obesity may cause multiple comorbidities and pose greater risks to human health. In addition, in present society, being overweight may lead to a person's low self-esteem and subsequent depressive symptoms, which in turn can have a negative impact on healthy aging (44). These mechanisms suggest that the overweight to obesity trajectory in older adults is an important marker of unhealthy aging and should be alerted.

## Strengths and limitations

This study has several advantages and limitations. Our results are based on a large national cohort of older adults in a developing country with high response rates and high follow-up compliance; a prospective cohort design was used in this study; the results of the overall health status of the older adult population were assessed using a comprehensive assessment of healthy aging; the LCGM was used to identify the change trajectory of BMI over time; and detailed consideration of various potential confounders.

In interpreting our findings, we should be aware of these limitations. First, there may be measurement errors in measures such as height and weight, and measures of health outcomes are largely self-reported, with potential recall bias issues as with other studies using self-rated results (10, 17), but repeated measurements have attempted to address this limitation. Second, the duration of follow-up was too short, so reverse causation may still be unavoidable. In addition, due to limitations in data availability, this study was unable to obtain additional follow-up measurements of BMI, potentially leading to the oversight of more intriguing trends in BMI changes. Finally, this is an observational study, therefore, we cannot rule out unmeasurable residual confounding factors. Moreover, our participants are focused on the Chinese older adults, which may affect the generalizability of our findings to other ethnic populations. While our study minimizes the confounding of sociocultural-economic status, changes of BMI can vary by age, race, and other settings. Therefore, further studies in different populations are needed to confirm our findings.

## Conclusion

Our cohort study found that most Chinese older adults aged 60 and above had stable normal or low BMI. Compared to individuals with the normal slight increase trajectory, individuals with the overweight to obesity trajectory had a significantly lower odds of healthy aging, and a stronger association existed for the chronic disease dimension. Interestingly, we found that individuals in the low-normal stable trajectory were associated with lower odds of cognitive impairment. Therefore, this study suggests that maintaining a stable normal weight and avoiding excessive weight variation are key factors for healthy aging. These findings add new evidence to previous research on healthy aging and provide further scientific support for weight management suggestions, that is, maintaining a stable and normal weight can obtain long-term health benefits.

## Data Availability

The original contributions presented in the study are included in the article/Supplementary material, further inquiries can be directed to the corresponding author.
