# Supplementary material for: Association of BMI trajectories and healthy aging among Chinese older adults: a national cohort study
Source: Front Public Health. 2025 Jun 9;13:1538261. doi: 10.3389/fpubh.2025.1538261 (PMC12183058; doi:10.3389/fpubh.2025.1538261)
Supplement: Supplementary file 1 [file Data_Sheet_1.docx]

**Association of BMI trajectories and healthy aging among Chinese older adults: a national cohort study**

**Supplementary materials**

Appendix 1 Sensitivity analyses 2

Appendix 2 Results of sensitivity analyses 3

eTable 1 Age-adjusteda baseline characteristics of included and excludedb participants due to missing healthy aging data 4

eTable 2 Fit indices of latent class growth modelling on BMI trajectories 5

eTable 3 *ORs* (95%*CIs*) of healthy aging by BMI trajectories by excluding participants who died between 2014 and 2018 (*n*=1,647) 6

eTable 4 *ORs* (95%*CIs*) of healthy aging with different standard deviation of BMI 7

eTable 5 *ORs* (95%*CIs*) of healthy aging and its four domains by different BMI in 2014 and standard deviation of BMI 8

eFigure 1 Selection of participants for the study. 10

eFigure 2 Restrictive cubic spline curve of BMI (2014) and the four dimensions of healthy aging. 11

eFigure 3 Association between standard deviation of BMI from 2008 to 2014 and healthy aging in 2018. (n=2,698) 12

Appendix 1 Sensitivity analyses

For verifying the robustness of the results, we conducted the following sensitivity analyses: (1) To evaluate the association between BMI trajectories and healthy aging among survivors, we excluded the older adults who died before the end of follow-up from the normal age group and then repeated all analyses; (2) In order to further control the confounding of social demography characteristics, we restricted the participants to farmers, illiterate people, Han people, and people with adequate living expenses, and reassessed the association of BMI trajectories with healthy aging; (3) Previous studies had found that hypertension and self-rated health status are predictive indicators of mortality and health outcomes, so we further adjusted these two variables ^[1](#_ENREF_1), [2](#_ENREF_2)^; (4) As far as we know, since the definition of healthy aging has not yet been uniformly defined in China, according to the relevant literature, we added the assessment of leisure activities or IADL to the current definition and explored the association between BMI trajectories and the new healthy aging ^[3](#_ENREF_3), [4](#_ENREF_4)^.

In addition to BMI trajectory, we conducted a secondary analysis, which analyzed the association between BMI variability and healthy aging from another perspective (standard deviation of BMI).

To investigate the potential mediating mechanisms of the association between overweight to obesity trajectory and healthy aging, we conducted causal mediation analysis using SAS PROC CAUSALMED^[5](#_ENREF_5)^. We explored a range of potential mediators (e.g., systolic blood pressure, diastolic blood pressure, C-reactive protein, physical activity). The results will be presented as average causal mediation effects (ACME), average direct effects (ADE), and total effects.

Appendix 2 Results of sensitivity analyses

**Sensitivity analyses**

We performed several sensitivity analyses. As shown in **Table 4**, firstly, we observed similar results when the analysis was limited to farmers, Han Chinese, illiterate, have adequate living expenses. Secondly, additional adjustments for hypertension or self-rated health status also did not substantially change our results. Thirdly, as some studies indicated that both leisure activities and IADL may be part of the assessment of healthy aging, we redefined healthy aging and found that the association between BMI trajectories and newly defined healthy aging was consistent. We subsequently excluded 1,051 participants who died between 2014 and 2018 and repeated the above analyses (*n*=1,647) and found that the main results remained similar ( **eTable 3**).

**Association between three-wave BMI variability and healthy aging**

In the secondary analysis, we used the standard deviation of BMI (BMI_SD_) to explore association between BMI variability and healthy aging from another perspective. **eFigure 3** illustrated the larger variability in BMI and the lower rate of healthy aging. After adjusting for all potential confounders, compared with individuals in the 1st quartile of BMI_SD_, the odds of healthy aging was significantly lower in the 4th quartile of BMI_SD_ (*OR*=0.57, 95%*CI*: 0.42-0.78) (**eTable 4**).

**Mediation Analysis**

In causal mediation analysis, we did not find any significant results, but the associations of overweight to obesity trajectory with chronic disease dimension of healthy aging were particularly notable. Thus, we speculate that BMI may be a direct effect of healthy aging.

eTable 1 Age-adjusted ^a^ baseline characteristics of included and excluded ^b^ participants due to missing healthy aging data

| Characteristic | **Excluded (**n=1,036) | **Included (**n=2,698) | P-value |
| --- | --- | --- | --- |
| Age (median (IQR)), years ^c^ | 77.00 (70.00, 86.00) | 78.00 (71.00, 86.00) | 0.150 |
| Female, *n* (%) | 562 (55.11) | 1398 (52.94) | 0.234 |
| Ethnic group, Han, *n* (%) | 986 (95.24) | 2494 (92.29) | **0.001** |
| Residential district, *n* (%) |  |  | <0.001 |
| Rural | 642 (61.49) | 1871 (69.30) |  |
| Town | 206 (19.95) | 582 (21.62) |  |
| City | 188 (18.56) | 245 (9.08) |  |
| Education level, *n* (%) ^d^ |  |  | **0.002** |
| Illiteracy | 535 (53.55) | 1468 (56.46) |  |
| Primary school and below | 346 (32.29) | 950 (33.85) |  |
| Junior high school and above | 154 (14.06) | 278 (9.60) |  |
| Marital status, *n* (%) |  |  | 0.227 |
| Married and living with spouse | 525 (47.82) | 1307 (45.62) |  |
| Others | 511 (52.18) | 1391 (54.38) |  |
| Farmer, *n* (%) ^d^ | 679 (65.55) | 2078 (77.24) | <0.001 |
| Living expenses, adequate, *n* (%) | 816 (78.83) | 2103 (77.88) | 0.531 |
| Smoking status, *n* (%) |  |  | 0.298 |
| Never | 684 (66.98) | 1713 (64.31) |  |
| Former | 117 (11.08) | 333 (12.30) |  |
| Current | 235 (21.94) | 652 (23.39) |  |
| Drinking status, *n* (%) |  |  | 0.480 |
| Never | 692 (67.23) | 1741 (65.13) |  |
| Former | 119 (11.44) | 324 (12.05) |  |
| Current | 225 (21.33) | 633 (22.81) |  |
| Exercise status, *n* (%) |  |  | <0.001 |
| Never | 561 (54.25) | 1640 (60.99) |  |
| Former | 94 (9.33) | 225 (8.42) |  |
| Current | 381 (36.42) | 833 (30.59) |  |
| Dietary diversity, good, *n* (%) | 802 (76.54) | 1971 (72.52) | **0.013** |
| Sleep duration, hours, *n* (%) |  |  | **0.014** |
| <7 | 242 (23.20) | 715 (26.36) |  |
| 7-8 | 496 (47.27) | 1152 (42.08) |  |
| >8 | 298 (29.53) | 831 (31.56) |  |
| Sleep quality, *n* (%) |  |  | 0.436 |
| Good | 706 (68.13) | 1835 (68.06) |  |
| Fair | 247 (23.79) | 611 (22.64) |  |
| Bad | 83 (8.08) | 252 (9.29) |  |
| Body mass index, kg/m^2^, mean (SD) | 20.97 (3.30) | 20.74 (3.31) | 0.055 |

Results in the table: the value of a continuous variable is the mean (SD)/a categorical variables is the *n* (%). The Chi-square test was used for comparing unordered categorical data and the Kruskal-Wallis test was used for ordinal data and continuous skewed data, and bold values indicated statistical significance *P* < 0.05.

^a^ Data were normalized according to the age distribution (continuous variable) of the study population in 2008.

^b^ Excluded for missing information related to healthy aging in 2018.

^c^ Value was not age adjusted.

^d^ The missing values for occupation and education were 4 and 3, respectively.

**eTable 2** Fit indices of latent class growth modeling on BMI trajectories

| Model | Log likelihood | AIC | BIC | aBIC | Entropy | Class (%) |
| --- | --- | --- | --- | --- | --- | --- |
| **1** | -21686.80 | 43379.60 | 43397.30 | 43387.77 | 1.00 | 100 |
| **2** | -20780.23 | 41572.47 | 41607.87 | 41588.81 | 0.77 | 24.68/ 75.32 |
| **3** | -20479.63 | 40977.27 | 41030.37 | 41001.78 | 0.73 | **49.30/ 43.07/7.64** |
| **4** | -20422.63 | 40869.25 | 40940.06 | 40901.93 | 0.70 | 47.15/ 36.51/ 2.78/ 13.57 |
| **5** | -20399.09 | 40828.17 | 40916.68 | 40869.02 | 0.66 | 24.35/6.08/46.03/0.67/22.87 |
| **6** | -20361.52 | 40759.04 | 40865.24 | 40808.05 | 0.69 | 0.85/22.46/1.04/6.12/45.14/24.39 |
| **7** | -20353.96 | 40749.92 | 40873.82 | 40807.10 | 0.70 | 1.89/0.22/0.92/19.90/44.26/26.24/6.56 |

Abbreviations: AIC, Akaike Information Criterion; BIC, Bayesian Information Criterion; aBIC, adjusted Bayesian Information Criterion.

eTable 3 *ORs* (95%*CIs*) of healthy aging by BMI trajectories by excluding participants who died between 2014 and 2018 (*n*=1,647)

| **Outcomes** | **No.** | **Healthy aging, *n* (%)** | **Model Ⅰ-Adjusted ^a^** | **Model Ⅱ-Adjusted ^b^** | **Model Ⅲ-Adjusted ^c^** |
| --- | --- | --- | --- | --- | --- |
| **Healthy aging by BMI trajectories** | | |  |  |  |
| Low-normal stable | 705 | 215 (30.50) | 1.03 (0.82, 1.29) | 1.00 (0.80, 1.26) | 1.03 (0.79, 1.34) |
| Normal slight increase | 788 | 260 (32.99) | 1.00 [Reference] | 1.00 [Reference] | 1.00 [Reference] |
| Overweight to obesity | 154 | 43 (27.92) | **0.67 (0.45, 0.99)** | 0.67 (0.45, 1.00) | **0.57 (0.34, 0.95)** |
| **Free of main chronic diseases by BMI trajectories** | | |  |  |  |
| Low-normal stable | 705 | 428 (60.71) | **1.25 (1.01, 1.54)** | 1.18 (0.96, 1.46) | 1.24 (0.98, 1.58) |
| Normal slight increase | 788 | 432 (54.82) | 1.00 [Reference] | 1.00 [Reference] | 1.00 [Reference] |
| Overweight to obesity | 154 | 64 (41.56) | **0.61 (0.43, 0.87)** | **0.65 (0.46, 0.93)** | **0.45 (0.29, 0.72)** |
| **No physical limitations by BMI trajectories** | | |  |  |  |
| Low-normal stable | 705 | 527 (74.75) | 1.28 (0.99, 1.65) | 1.22 (0.94, 1.58) | 1.00 (0.74, 1.34) |
| Normal slight increase | 788 | 596 (75.63) | 1.00 [Reference] | 1.00 [Reference] | 1.00 [Reference] |
| Overweight to obesity | 154 | 121 (78.57) | 0.86 (0.55, 1.33) | 0.94 (0.60, 1.47) | 1.33 (0.76, 2.33) |
| **Good mental health by BMI trajectories** | | |  |  |  |
| Low-normal stable | 705 | 395 (56.03) | 0.86 (0.70, 1.07) | 0.92 (0.74, 1.14) | 1.05 (0.82, 1.34) |
| Normal slight increase | 788 | 493 (62.56) | 1.00 [Reference] | 1.00 [Reference] | 1.00 [Reference] |
| Overweight to obesity | 154 | 102 (66.23) | 1.02 (0.70, 1.48) | 0.92 (0.63, 1.35) | 0.82 (0.51, 1.33) |
| **No cognitive impairment by BMI trajectories** | | |  |  |  |
| Low-normal stable | 705 | 516 (73.19) | 0.80 (0.61, 1.05) | 0.80 (0.61, 1.05) | 0.75 (0.55, 1.02) |
| Normal slight increase | 788 | 640 (81.22) | 1.00 [Reference] | 1.00 [Reference] | 1.00 [Reference] |
| Overweight to obesity | 154 | 135 (87.66) | 1.12 (0.65, 1.92) | 1.09 (0.63, 1.87) | 1.17 (0.60, 2.29) |

Abbreviations: BMI, body mass index (calculated as weight (kg) divided by height (meters) squared); *OR*, odds ratio; *CI*, confidence interval.

Bold values indicated statistical significance *P* < 0.05.

^a^ Model I was adjusted for age (continuous), gender (male, female).

^b^ Model Ⅱ was additionally adjusted for residence district (city, town, rural); ethnicity (Han, others); educational level (illiteracy, primary school and below, junior high school and above); marital status (married and living with spouse, others); living expenses (adequate, inadequate); occupation (farmer, others).

^c^ Model Ⅲ was additionally adjusted for lifestyle factors, including smoking history (never, former, current); drinking status (never, former, current); exercise (never, former, current); dietary diversity (good, bad); sleep quality (good, fair, bad) and sleep duration (<7, 7-8, >8 hours), and BMI at baseline (underweight, normal, overweight, obesity).

eTable 4 *ORs* (95%*CIs*) of healthy aging with different standard deviation of BMI

| **Outcomes** | **Standard deviation of BMI data measured by three waves, *ORs* (95%*CIs*)** | | | | |
| --- | --- | --- | --- | --- | --- |
|  | **Q1**  **(0.00, 1.03 kg/m^2^)** | **Q2**  **(1,04, 1.75 kg/m^2^)** | | **Q3**  **(1.76, 2.66 kg/m^2^)** | **Q4**  **(≥2.67 kg/m^2^)** |
| **Healthy agin*g, n (%)*** | 164 (24.62) | 148 (21.76) | 120 (17.83) | | 86 (12.67) |
| **Healthy aging** | 1.00 [Reference] | 0.89 (0.68, 1.17) | 0.78 (0.59, 1.04) | | **0.57 (0.42, 0.78)** |

Abbreviations: *ORs*, odds ratios; *CIs*, confidence intervals; BMI, body mass index (calculated as: weight (kg) divided by height (meters) squared).

Q1-Q4 was divided equally by quartile.

Model was adjusted for age (continuous), gender (male, female); residence (city, town, rural); ethnic group (Han, others); educational level (illiteracy, primary school and below, junior high school and above); marital status (married and living with spouse, others); living expenses (adequate, inadequate); occupation(farmer, others); and lifestyle factors, including smoking history (never, former smoker, or current smoker); drinking status (never, former, or current); exercise(never, former, or current); dietary diversity (good, bad); sleep quality (good, fair, bad) and sleep duration (<7, 7-8, >8 hours), and BMI at baseline (underweight, normal, overweight, obesity).

eTable 5 *ORs* (95%*CIs*) of healthy aging **and its four domains** by different BMI in 2014 and standard deviation of BMI

| **Outcome** | **No.** | **Healthy aging, *n* (%)** | **Model l ^a^** | **Model Ⅱ ^b^** | **Model Ⅲ ^c^** |
| --- | --- | --- | --- | --- | --- |
| **Healthy aging by BMI** | | | | | |
| **BMI in 2014** |  |  |  |  |  |
| <18.5 | 544 | 69 (12.68) | 0.79 (0.59, 1.07) | 0.79 (0.58, 1.06) | 0.85 (0.63, 1.15) |
| 18.5- | 1,520 | 316 (20.79) | 1.00 [Reference] | 1.00 [Reference] | 1.00 [Reference] |
| 24- | 489 | 100 (20.45) | **0.76 (0.58, 1.00)** | 0.78 (0.59, 1.02) | 0.78 (0.59, 1.02) |
| 28- | 145 | 33 (22.76) | 0.74 (0.48, 1.13) | 0.76 (0.49, 1.17) | 0.75 (0.48, 1.17) |
| *P* for trend | 2,698 | 518 (19.20) | 0.339 | 0.449 | 0.279 |
| **Continuous, per 1 kg/m^2^** | 2,698 | 518 (19.20) | 0.99 (0.96, 1.02) | 0.99 (0.96, 1.02) | 0.99 (0.96, 1.02) |
| **BMI_SD_** |  |  |  |  |  |
| Q1 | 666 | 164 (24.62) | 1.00 [Reference] | 1.00 [Reference] | 1.00 [Reference] |
| Q2 | 680 | 148 (21.76) | 0.89 (0.68, 1.16) | 0.90 (0.69, 1.18) | 0.90 (0.68, 1.18) |
| Q3 | 673 | 120 (17.83) | 0.80 (0.60, 1.06) | 0.81 (0.61, 1.07) | 0.81 (0.61, 1.09) |
| Q4 | 679 | 86 (12.67) | **0.57 (0.42, 0.77)** | **0.59 (0.43, 0.80)** | **0.61 (0.44, 0.83)** |
| *P* for trend | 2,698 | 518 (19.20) | **<0.001** | **<0.001** | **0.002** |
| **Continuous, per 1 kg/m^2^** | 2,698 | 518 (19.20) | **0.86 (0.79, 0.94)** | **0.86 (0.79, 0.94)** | **0.87 (0.80, 0.95)** |
| **Free of main chronic diseases by BMI** | | | | | |
| **BMI in 2014** |  |  |  |  |  |
| <18.5 | 544 | 141 (25.92) | **0.72 (0.57, 0.90)** | **0.71 (0.57, 0.90)** | **0.75 (0.59, 0.94)** |
| 18.5- | 1,520 | 565 (37.17) | 1.00 [Reference] | 1.00 [Reference] | 1.00 [Reference] |
| 24- | 489 | 175 (35.79) | **0.79 (0.64, 0.99)** | 0.82 (0.66, 1.03) | 0.83 (0.66, 1.04) |
| 28- | 145 | 47 (32.41) | **0.59 (0.41, 0.86)** | **0.62 (0.42, 0.90)** | **0.63 (0.43, 0.92)** |
| *P* for trend | 2,698 | 928 (34.40) | 0.421 | 0.663 | 0.524 |
| **Continuous, per 1 kg/m^2^** | 2,698 | 928 (34.40) | 1.00 (0.97, 1.02) | 1.00 (0.98, 1.02) | 1.00 (0.97, 1.02) |
| **BMI_SD_** |  |  |  |  |  |
| Q1 | 666 | 260 (39.04) | 1.00 [Reference] | 1.00 [Reference] | 1.00 [Reference] |
| Q2 | 680 | 222 (32.65) | **0.76 (0.60, 0.95)** | **0.76 (0.61, 0.96)** | **0.77 (0.61, 0.97)** |
| Q3 | 673 | 245 (36.40) | 1.00 (0.80, 1.26) | 1.00 (0.79, 1.26) | 1.02 (0.80, 1.29) |
| Q4 | 679 | 201 (29.60) | **0.76 (0.60, 0.97)** | **0.76 (0.60, 0.97)** | 0.79 (0.62, 1.00) |
| *P* for trend | 2,698 | 928 (34.40) | 0.166 | 0.159 | 0.270 |
| **Continuous, per 1 kg/m^2^** | 2,698 | 928 (34.40) | **0.93 (0.87, 1.00)** | **0.93 (0.87, 1.00)** | 0.94 (0.88, 1.00) |
| **Good physical function by BMI** | | | | | |
| **BMI in 2014** |  |  |  |  |  |
| <18.5 | 544 | 181 (33.27) | 0.81 (0.64, 1.03) | 0.81 (0.63, 1.03) | 0.83 (0.65, 1.06) |
| 18.5- | 1,520 | 724 (47.63) | 1.00 [Reference] | 1.00 [Reference] | 1.00 [Reference] |
| 24- | 489 | 267 (54.60) | 0.97 (0.76, 1.23) | 0.97 (0.76, 1.23) | 0.97 (0.76, 1.23) |
| 28- | 145 | 77 (53.10) | **0.65 (0.44, 0.96)** | **0.66 (0.45, 0.98)** | 0.67 (0.45, 1.00) |
| *P* for trend | 2,698 | 1,249 (46.29) | 0.816 | 0.860 | 0.808 |
| **Continuous, per 1 kg/m^2^** | 2,698 | 1,249 (46.29) | 1.00 (0.97, 1.02) | 1.00 (0.97, 1.02) | 1.00 (0.97, 1.02) |
| **BMI_SD_** |  |  |  |  |  |
| Q1 | 666 | 342 (51.35) | 1.00 [Reference] | 1.00 [Reference] | 1.00 [Reference] |
| Q2 | 680 | 342 (50.29) | 0.99 (0.77, 1.27) | 1.02 (0.80, 1.31) | 1.03 (0.80, 1.33) |
| Q3 | 673 | 317 (47.10) | 1.13 (0.88, 1.46) | 1.15 (0.89, 1.49) | 1.15 (0.89, 1.48) |
| Q4 | 679 | 248 (36.52) | **0.72 (0.56, 0.93)** | **0.74 (0.57, 0.96)** | **0.76 (0.58, 0.98)** |
| *P* for trend | 2,698 | 1,249 (46.29) | **0.037** | 0.061 | 0.084 |
| **Continuous, per 1 kg/m^2^** | 2,698 | 1,249 (46.29) | **0.89 (0.83, 0.96)** | **0.89 (0.83, 0.96)** | **0.90 (0.84, 0.96)** |
| **Good mental health by BMI** | | | | | |
| **BMI in 2014** |  |  |  |  |  |
| <18.5 | 544 | 123 (22.61) | **0.64 (0.50, 0.81)** | **0.66 (0.51, 0.85)** | **0.71 (0.55, 0.92)** |
| 18.5- | 1,520 | 580 (38.16) | 1.00 [Reference] | 1.00 [Reference] | 1.00 [Reference] |
| 24- | 489 | 223 (45.60) | 1.08 (0.87, 1.36) | 1.02 (0.81, 1.28) | 1.00 (0.79, 1.27) |
| 28- | 145 | 68 (46.90) | 0.92 (0.64, 1.34) | 0.85 (0.59, 1.25) | 0.86 (0.58, 1.27) |
| *P* for trend | 2,698 | 994 (36.84) | **0.008** | 0.063 | 0.168 |
| **Continuous, per 1 kg/m^2^** | 2,698 | 994 (36.84) | **1.03 (1.01, 1.06)** | 1.02 (1.00, 1.05) | 1.02 (0.99, 1.04) |
| **BMI_SD_** |  |  |  |  |  |
| Q1 | 666 | 287 (43.09) | 1.00 [Reference] | 1.00 [Reference] | 1.00 [Reference] |
| Q2 | 680 | 264 (38.82) | 0.85 (0.67, 1.07) | 0.87 (0.69, 1.11) | 0.88 (0.68, 1.12) |
| Q3 | 673 | 248 (36.85) | 0.94 (0.74, 1.20) | 1.00 (0.78, 1.28) | 1.03 (0.80, 1.32) |
| Q4 | 679 | 195 (28.72) | **0.67 (0.53, 0.86)** | **0.73 (0.57, 0.94)** | **0.76 (0.59, 0.99)** |
| *P* for trend | 2,698 | 994 (36.84) | **0.008** | 0.052 | 0.126 |
| **Continuous, per 1 kg/m^2^** | 2,698 | 994 (36.84) | **0.89 (0.83, 0.96)** | **0.91 (0.85, 0.98)** | **0.92 (0.86, 0.99)** |
| **Good cognitive function by BMI** | | | | | |
| **BMI in 2014** |  |  |  |  |  |
| <18.5 | 544 | 168 (30.88) | **0.66 (0.52, 0.84)** | **0.66 (0.52, 0.85)** | **0.69 (0.53, 0.88)** |
| 18.5- | 1,520 | 740 (48.68) | 1.00 [Reference] | 1.00 [Reference] | 1.00 [Reference] |
| 24- | 489 | 294 (60.12) | 1.22 (0.95, 1.55) | 1.19 (0.93, 1.52) | 1.19 (0.93, 1.53) |
| 28- | 145 | 94 (64.83) | 1.11 (0.73, 1.67) | 1.08 (0.71, 1.63) | 1.12 (0.74, 1.71) |
| *P* for trend | 2,698 | 1,296 (48.04) | **<0.001** | **<0.001** | **0.001** |
| **Continuous, per 1 kg/m^2^** | 2,698 | 1,296 (48.04) | **1.05 (1.03, 1.08)** | **1.05 (1.02, 1.08)** | **1.05 (1.02, 1.08)** |
| **BMI_SD_** |  |  |  |  |  |
| Q1 | 666 | 355 (53.30) | 1.00 [Reference] | 1.00 [Reference] | 1.00 [Reference] |
| Q2 | 680 | 350 (51.47) | 0.93 (0.75, 1.15) | 0.94 (0.73, 1.22) | 0.96 (0.75, 1.25) |
| Q3 | 673 | 319 (47.40) | **0.79 (0.64, 0.98)** | 1.04 (0.80, 1.34) | 1.08 (0.84, 1.41) |
| Q4 | 679 | 272 (40.06) | **0.59 (0.47, 0.73)** | 0.79 (0.61, 1.02) | 0.83 (0.64, 1.08) |
| *P* for trend | 2,698 | 1,296 (48.04) | **<0.001** | 0.140 | 0.284 |
| **Continuous, per 1 kg/m^2^** | 2,698 | 1,296 (48.04) | **0.85 (0.80, 0.90)** | **0.93 (0.86, 0.99)** | 0.93 (0.87, 1.00) |

^a^ Model I was adjusted for age (continuous), gender (male, female).

^b^ Model Ⅱ was additionally adjusted for residence (city, town, rural); ethnicity (Han, others); educational level (illiteracy, primary school and below, junior high school and above); marital status (married and living with spouse, others); living expenses (adequate, inadequate); occupation (farmer, others).

^c^ Model Ⅲ was additionally adjusted for lifestyle factors, including smoking history (never, former, current); drinking status (never, former, current); exercise (never, former, current); dietary diversity (good, bad); sleep quality (good, fair, bad) and sleep duration (<7, 7-8, >8 hours).


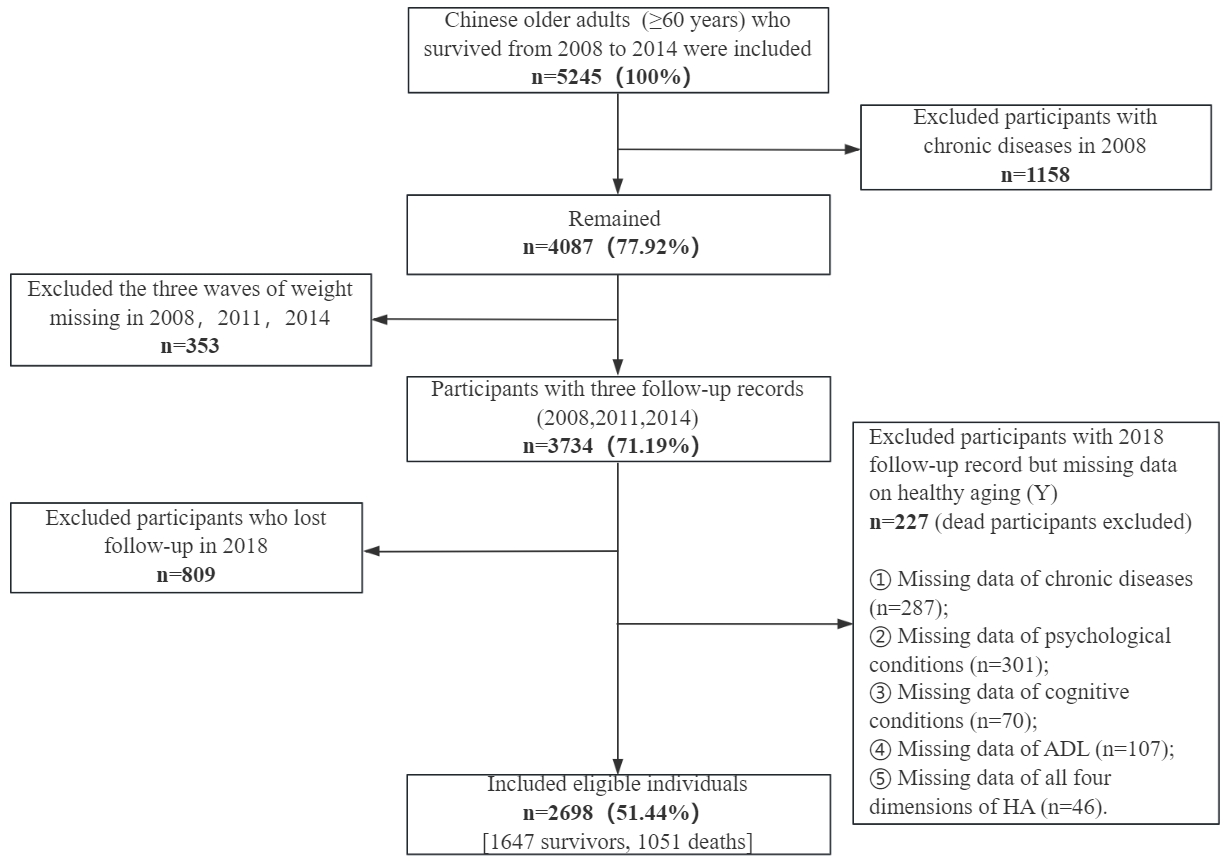


**eFigure** 1 Selection of participants for the study.

**
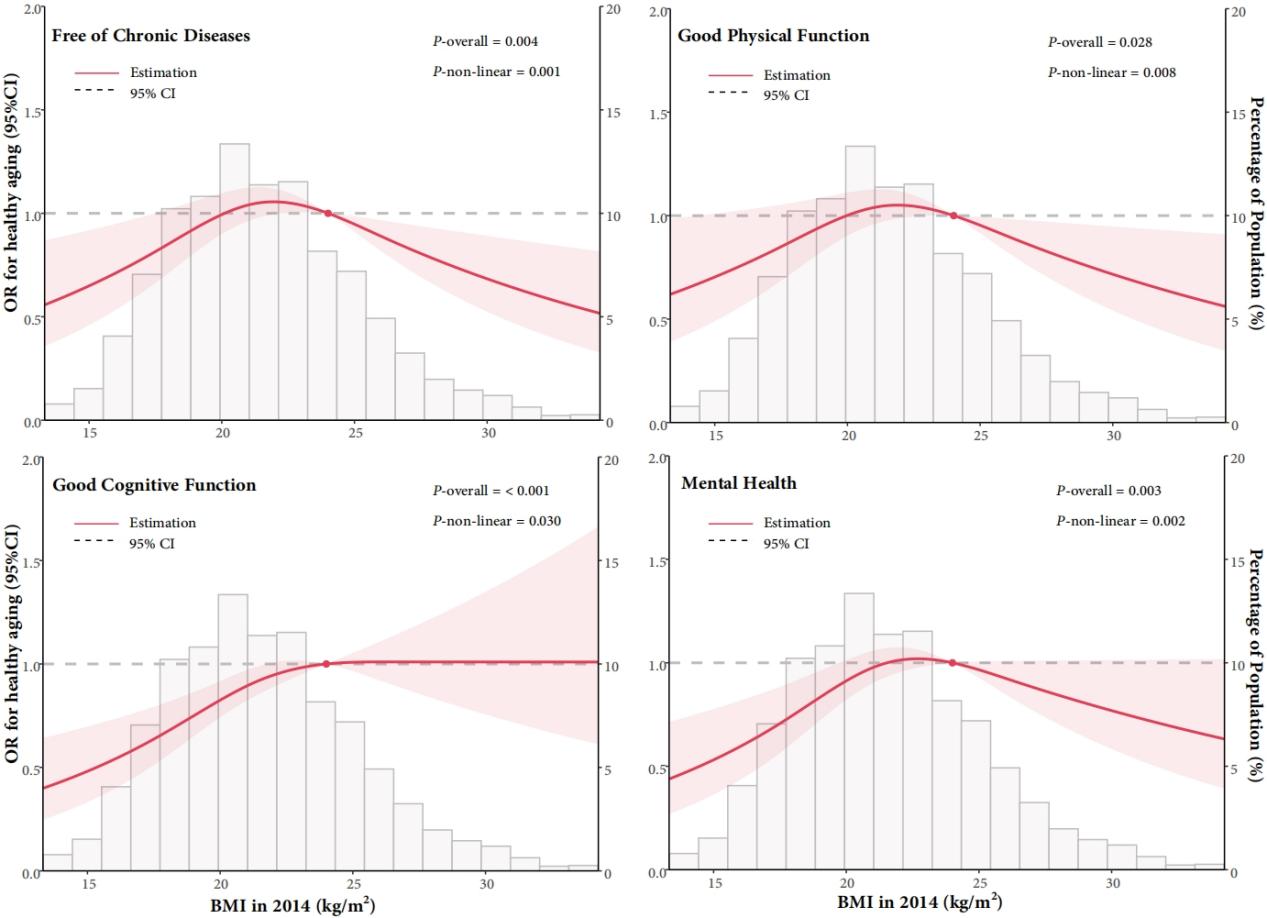
**

eFigure 2 Restrictive cubic spline curve of BMI (2014) and the four dimensions of healthy aging.

Adjusted for age (continuous), gender (male, female); residence (city, town, rural); ethnic group (Han, others); educational level (illiteracy, primary school and below, junior high school and above); marital status (married and living with spouse, others); living expenses (adequate, inadequate); occupation(farmer, others); and lifestyle factors, including smoking history (never, former smoker, or current smoker); drinking status (never, former, or current); exercise(never, former, or current); dietary diversity (good, bad); sleep quality (good, fair, bad) and sleep duration (<7, 7-8, >8 hours).

The optimal threshold for BMI is about 22 kg/m^2^ for chronic disease, 22 kg/m^2^ for physical function, 24 kg/m^2^ for cognitive function, and 23 kg/m^2^ for mental health.


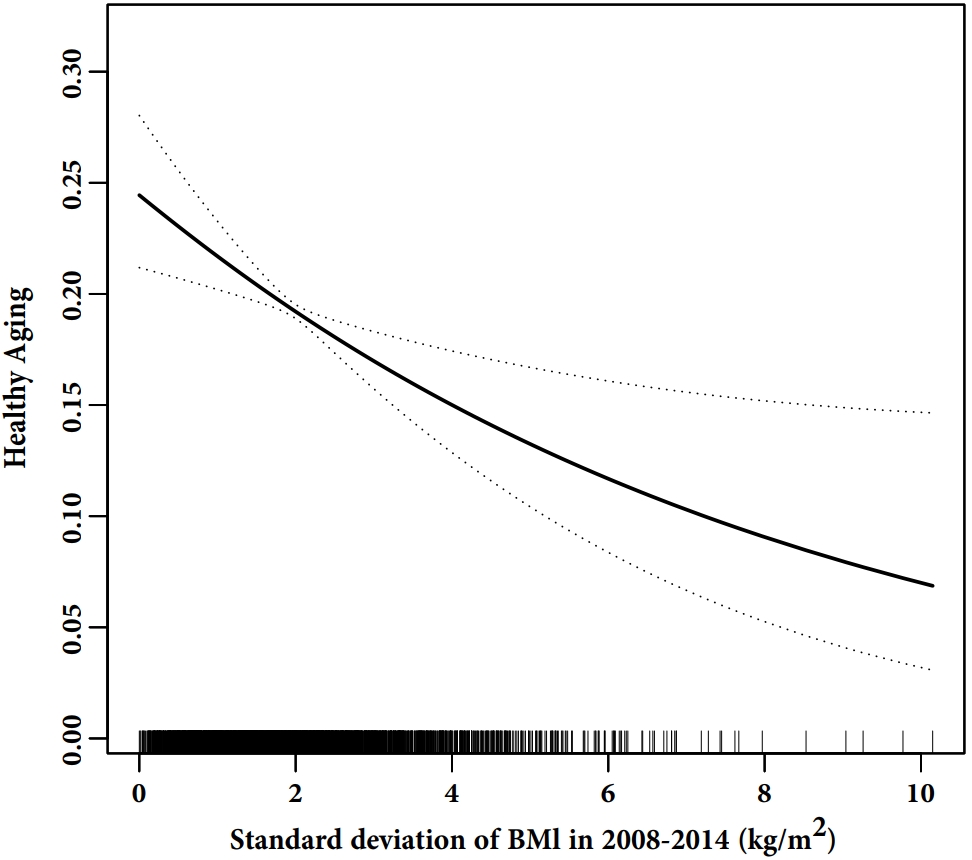


**eFigure 3** Association between standard deviation of BMI from 2008 to 2014 and healthy aging in 2018. (n=2,698)

Model was adjusted for age (continuous), gender (male, female); residence (city, town, rural); ethnic group (Han, others); educational level (illiteracy, primary school and below, junior high school and above); marital status (married and living with spouse, others); living expenses (adequate, inadequate); occupation(farmer, others); and lifestyle factors, including smoking history (never, former smoker, or current smoker); drinking status (never, former, or current); exercise(never, former, or current); dietary diversity (good, bad); sleep quality (good, fair, bad) and sleep duration (<7, 7-8, >8 hours), and BMI at baseline (underweight, normal, overweight, obesity).

**Supplementary References:Uncategorized References**

**1.** Zeng Y, Chen H, Shi X, et al. Health consequences of familial longevity influence among the Chinese elderly. *J Gerontol A Biol Sci Med Sci.* 2013;68(4):473-482. <https://www.ncbi.nlm.nih.gov/pubmed/23064818>.

**2.** Ruan R, Feng L, Li J, et al. Tea consumption and mortality in the oldest-old Chinese. *J Am Geriatr Soc.* 2013;61(11):1937-1942. <https://www.ncbi.nlm.nih.gov/pubmed/24117374>.

**3.** Zhang Y, Xiong Y, Yu Q, et al. The activity of daily living (ADL) subgroups and health impairment among Chinese elderly: a latent profile analysis. *BMC Geriatr.* 2021;21(1):30. <https://www.ncbi.nlm.nih.gov/pubmed/33413125>.

**4.** Gong J, Wang G, Wang Y, et al. Nowcasting and forecasting the care needs of the older population in China: analysis of data from the China Health and Retirement Longitudinal Study (CHARLS). *Lancet Public Health.* 2022. <https://www.ncbi.nlm.nih.gov/pubmed/36423656>.

**5.** YF Y, M L, W Z. Causal mediation analysis with the CAUSALMED procedure. *SAS Global Forum, Cary, NC, USA. SAS Institute Inc.* 2018. https:// www. sas. com/ conte nt/ dam/ SAS/ suppo rt/ en/ sas- globalforum- proce edings/ 2018/ 1991- 2018. pdf.
